# Supplementary material for: Body size and life history shape the historical biogeography of tetrapods
Source: Nat Ecol Evol. 2023 Aug 21;7(9):1467–79. doi: 10.1038/s41559-023-02150-5 (PMC10482685; doi:10.1038/s41559-023-02150-5)
Supplement: Supplementary file 1 — Supplementary methods, discussion and Table 2. [file 41559_2023_2150_MOESM1_ESM.pdf]

nature ecology & evolution

Article https://doi.org/10.1038/s41559-023-02150-5

Body size and life history shape the historical biogeography of tetrapods

In the format provided by the authors and unedited

Supplementary Information to Uncovering how body size and life history have shaped the historical biogeography of tetrapods

Phylogenetic uncertainty

We assessed the effect of phylogenetic uncertainty on the classification of trait-dispersal relationships by rerunning analyses on a selection of clades (one clade per class). For reptiles, amphibians and mammals we selected a clade at random (Natricinae, Hynobiidae, Sciuridae), and extracted ten phylogenies from their respective posteriors[11]. For the bird clade (Pycnonotidae) we repeated analyses on a phylogeny where we used the backbone of Prum et al.[50] that included the fossil vegavis. For each clade and phylogeny, we (1) repositioned species on the body size and life history axes using phylogenetic factor analyses (PFAs). We then (2) repeated all biogeographic models, using the same base models and manual dispersal multiplier matrices as for the main analyses (Extended Data Tab. 1), and (3) reclassified trait-dispersal relationships. For Hynobiidae, Sciuridae and Pycnonotidae, trait-dispersal relationships varied little (Extended Data Fig. 4, Extended Data Tab. 4). However, there was considerable variation for Natricinae. The main analysis identified a positive relationship between body size and dispersal rates in Natricinae, i.e. large species were better dispersers than small ones. However, we identified the same body size-dispersal relationship in only five of the selected trees from the posterior, in three trees we identified intermediately-sized species to be better dispersers, and in two trees the difference in dispersal rates between most-dispersive and least-dispersive species was less than 10%. The results were similar for life history-dispersal relationships in Natricinae.

The trees selected from the posterior for Natricinae were more different than those of any of the other clades (minimum correlation Natricinae: 0.945; for other clades: 0.9804, function cor.dendlist from R package dendextend v1.16.0[117]), which suggests more uncertainty in the phylogenetic estimations in this clade than in others. Consequently, species scores from the different PFAs were less strongly correlated for Natricinae than those of other clades (minimum correlation Natricinae: 0.42; for

other clades: 0.99). This indicated that we had difficulties identifying species' positions on the body size and life history axes which led to differences in categorizations of species as dispersal-prone and not dispersal-prone. This uncertainty in turn cascaded down to the biogeographic estimations and classifications of trait-dispersal relationships. In the present study, we analyse the global signal emerging from the responses of a large number of clades, and thus assume that using a single phylogeny per clade (with a potential clade-specific phylogenetic bias) generates noise to the overall signal. Adding noise should decrease the probability of detecting a signal, making our tests more conservative. In practice, the global character of this study was already computationally demanding (ca. 262800h*cores of calculations), and any replication for uncertainty analyses multiplies the computation time (and associated carbon emission), which we believe is not necessary.

Barriers in biogeographic dispersal

Barriers are species-specific: what constitutes a dispersal barrier for one species is not necessarily a barrier for another species. To define barriers as objectively as possible, we used a data-driven bioregionalisation approach, based on species' phylogenetic relationships and extant distribution data (see Weil et al.[11] for details concerning the methodology). Our bioregionalisation approach showed that for most clades continental barriers were stronger than oceanic ones. Since traits might be related in a similar manner to dispersal success across both types of barriers, we combined both barriers in our analyses. Large body size may be advantageous in oceanic dispersal because of increased stress tolerance[8], and it may be advantageous in continental dispersal because large-bodied species are generally better active dispersers than small species[6,32]. In both kinds of dispersal we expect founder populations to be small and life history effects should therefore be comparable, with either fast-lived species having advantage due to being more resistant to stochastic extinction[13,14,15], or slow-lived species having an advantage due to less demographic variability[16,17,18]. In addition, biogeographic dispersal is rare and difficult to observe. Combining both types of dispersal hence increased statistical power (if we had only included oceanic dispersal, the sample size would have been too small to statistically

detect the influence of traits on these scales since very few dispersal events would have been estimated on the phylogeny).

Combining trait databases

Amphibians: We used trait data from Allen et al.[66], which includes snout-vent-length (SVL, mm), clutch size (CS, number of eggs), clutches per year (CY), egg size (ES, mm), sexual maturity (SM, years) and reproductive lifespan (RL, years). We calculated longevity (LG, years) from SM and RL (LG = RL+SM). To this database we added body size (BS) data from Cooney & Thomas[67], maximal SVL, maximum CS and maximum LG from Trakimas et al.[68], and SVL and CS from Pincheira-Donoso et al.[69]. We inferred SVL values for species without SVL data based on BS data using phylogenetic linear models (R package phylolm v2.6[118]), separately for Caudata and all other amphibians. When adding data to Allen et al.[66], we first looked up synonyms in the Integrated Taxonomic Information System (ITIS; functions get_tsn and itis_getrecord, package taxize v0.9.99[57,58]) and AmphibiaWeb (2016; function synonymMatch, package rangeBuilder v1.5[59]) for all species in the new database. We also checked for outliers per order, using Tukey's fences where a value is considered an outlier if it is outside the bounds of [Q1 - 3*IQR, Q3+3*IQR], where Q1 and Q3 are the lower and upper quartiles, respectively, and IQR the inter-quartile range Q3-Q1. These calculations were done on logged trait values. Overall, we compiled SVL, ES, CS, SM and LG for amphibians.

Reptiles: We again used trait data from Allen et al.[66] as a starting point, which included SVL, hatchling SVL, CS, CY, SM (in months), LG, RL and hatchling body mass (HBM, g). If hatchling SVL was available but not HBM for a given species, we inferred HBM from hatchling SVL using phylogenetic regressions. We did this separately for Anguimorpha, Gekkota, Iguania, Lacertoidea and Scincoidea. We then added CS from Meiri et al.[70], BS from Cooney & Thomas[67], CS and CY from Schwarz & Meiri[71], SVL and BS from Feldman et al.[72], CS, CY, SM, LG and body mass (BM) from Myhrvold et al.[73], and CS, LG and BM from Stark et al.[74]. As for amphibians we first checked if synonyms of species in the new databases already existed in Allen et al.[66] (using ITIS and the reptile database[61]) and excluded outliers before adding values. We estimated

SVL from BS and BM measures where possible using phylogenetic regressions. Overall, we compiled SVL, HBM, CS, CY, SM and LG for non-avian reptiles.

Mammals: We combined data from PanTHERIA[75] (BM (g), gestation time (GT, days), litter size (LS), litters per year (LY), LG (months), SM (days) and weaning age (WA, days), neonate body mass (NBM, g)), Phylacine[76] (BW), AnAge[77] (BM, GT, LS, LY, LG, SM female, SM male, WA, NBM), Ernest[78] (BM, GT, LS, LY, LG, WA, NBM), Fisher et al.[79] (GT, LS, LG, SM female, WA, NBM), Myhrvold et al.[73] (BM, GT, LS, LY, LG, SM female, SM male, WA, NBM), Tsuboi et al.[80] (BM), Williams et al.[81] (BM, LS, LY, LG), Smith et al.[82] (BM), COMBINE[83] (BM, GT, LS, LY, LG, SM, SM female, SM male, WA) and Capellini et al.[84] (BM, GT, LS, LY, LG, SM, WA). Before combining the databases, we used the function getAcceptedNames (R package rangeBuilder) to update the taxonomies of the individual databases according to Wilson & Reeder[62]. From the AnAge database[77] we only used data from wild species that were flagged as "high" or "acceptable" data quality. We again checked for outliers per trait as described above. We also checked for outliers across databases if more than one database contained a value for a given trait and species. Where female sexual maturity was not available, we completed with male or unknown sexual maturity. When combining values across databases, we did a median of unique values, except for longevity, where we kept the maximum value. Overall, we compiled BM, NBM, LS, LY, LG, SM, GT and WA for mammals.

Birds: We combined data from Sheard et al.[85] (BM), Myhrvold et al.[73] (BM, CS, CY, LG, SM female, SM male, egg mass (EM), fledging age (FA)), Botero et al.[86] (BM), O'Gorman & Hone[87] (BM), Tsuboi et al.[80] (BM), AnAge[77] (BM, GT, CS, CY, LG, SM female, SM male), Garnett et al.[88] (BM, CS), Burgio et al.[89] (BM), Williams et al.[81] (BM, CS, CY, LG, SM), Dufour et al.[52] (BM), Storchová & Hořák[90] (BM, CS, CY, LG, SM), Tobias & Pigot[91] (CS), Rotenberry & Balasubramaniam[92] (EM) and DATLife[93] (SM, LG). Before combining the databases, we used the function getAcceptedNames (R package rangeBuilder) to update the individual databases according to the BirdLife Taxonomic Checklist (v8.0[62]). From the AnAge database[77] we only used data from wild species that were flagged as "high" or "acceptable" data quality. We excluded values of captive individuals from the DATLife database and only used SM of females. We excluded inferred values from Burgio et al.[89]. We again checked for outliers as described above per trait. We also checked for outliers across databases if more than

one database contained a value for a given trait and species. Where female sexual maturity was not available, we completed with male or unknown sexual maturity. When combining values across databases, we did a median of unique values, except for longevity, where we kept the maximum value. Overall, we compiled BM, EM, LS, LY, LG, SM, GT and WA for birds.

Supplementary Table 2 | Data coverage overview for class-level phylogenetic factor analyses.

For reptiles and amphibians: snout-vent-length, for mammals and birds: body mass. For amphibians: egg size, for birds: egg mass, for reptiles: hatchling body mass, for mammals: neonate body mass. Abbreviations: SVL/BM: snout-vent-length/body mass, HBM/ES: hatchling body mass/egg size, CS/LS: clutch/litter size, CY/LY: clutches/litters per year, SM: average age of females at sexual maturity, LG: maximum longevity, GT: gestation time, WA: weaning age/fledgling age.

Clade	Number of species included	SVL/BM	HBM/EM/ES	CS/LS	CY/LY	SM	LG	GT	WA
Amphibians	3447	3421 (99%)	401 (12%)	1621 (47%)	1267 (37%)	347 (10%)			
Reptiles	5657	5619 (99%)	2143 (38%)	3806 (67%)	1412 (25%)	963 (17%)	1563 (28%)		
Mammalia	3874	3874 (100%)	1538 (40%)	2794 (72%)	1844 (48%)	1675 (43%)	2121 (55%)	1847 (48%)	1692 (44%)
Aves	6589	6589 (100%)	3449 (52%)	6427 (98%)	1519 (23%)	1157 (18%)	1441 (22%)	1814 (28%)	1542 (23%)

References

117. Galili, T. dendextend: an R package for visualizing, adjusting, and comparing trees of hierarchical clustering. *Bioinformatics* **31**(22), 3718–3720 (2015).

118. Ho, L.S.T., & Ane, C. A linear-time algorithm for Gaussian and non-Gaussian trait evolution models. *Syst. Biol.* **63**, 397-408 (2014).
