# Supplementary material for: Body size and life history shape the historical biogeography of tetrapods
Source: Nat Ecol Evol. 2023 Aug 21;7(9):1467–79. doi: 10.1038/s41559-023-02150-5 (PMC10482685; doi:10.1038/s41559-023-02150-5)
Supplement: Supplementary file 2 — Reporting Summary [file 41559_2023_2150_MOESM2_ESM.pdf]

nature portfolio

Corresponding author(s): Sarah-Sophie Weil

Last updated by author(s): 12/06/2023

Reporting Summary

Nature Portfolio wishes to improve the reproducibility of the work that we publish. This form provides structure for consistency and transparency in reporting. For further information on Nature Portfolio policies, see our Editorial Policies and the Editorial Policy Checklist.

Statistics

For all statistical analyses, confirm that the following items are present in the figure legend, table legend, main text, or Methods section.

n/a	Confirmed	
☐	☒	The exact sample size (*n*) for each experimental group/condition, given as a discrete number and unit of measurement
☐	☒	A statement on whether measurements were taken from distinct samples or whether the same sample was measured repeatedly
☐	☒	The statistical test(s) used AND whether they are one- or two-sided<br>*Only common tests should be described solely by name; describe more complex techniques in the Methods section.*
☐	☒	A description of all covariates tested
☐	☒	A description of any assumptions or corrections, such as tests of normality and adjustment for multiple comparisons
☐	☒	A full description of the statistical parameters including central tendency (e.g. means) or other basic estimates (e.g. regression coefficient) AND variation (e.g. standard deviation) or associated estimates of uncertainty (e.g. confidence intervals)
☐	☒	For null hypothesis testing, the test statistic (e.g. *F*, *t*, *r*) with confidence intervals, effect sizes, degrees of freedom and *P* value noted<br>*Give P values as exact values whenever suitable.*
☒	☐	For Bayesian analysis, information on the choice of priors and Markov chain Monte Carlo settings
☒	☐	For hierarchical and complex designs, identification of the appropriate level for tests and full reporting of outcomes
☒	☐	Estimates of effect sizes (e.g. Cohen's *d*, Pearson's *r*), indicating how they were calculated

Our web collection on statistics for biologists contains articles on many of the points above.

Software and code

Policy information about availability of computer code

Data collection	No software was used to collect data. R v3.6.3 (<www.r-project.org>) was used to compile different databases
Data analysis	R v3.6.2 (<www.r-project.org>) was used for biogeographic analyses on the high-performance computation cluster, R v3.6.3 (<www.r-project.org>) was used for all further analyses. Data and code to support our results are deposited on Figshare. A doi is included in the data and code availability statements in the main manuscript file.

For manuscripts utilizing custom algorithms or software that are central to the research but not yet described in published literature, software must be made available to editors and reviewers. We strongly encourage code deposition in a community repository (e.g. GitHub). See the Nature Portfolio guidelines for submitting code & software for further information.

Data

Policy information about availability of data

All manuscripts must include a data availability statement. This statement should provide the following information, where applicable:

- Accession codes, unique identifiers, or web links for publicly available datasets
- A description of any restrictions on data availability
- For clinical datasets or third party data, please ensure that the statement adheres to our policy

Data for analyses of historical biogeography (trait-dependent and otherwise), as well as data related to the analysis of differences in the magnitude of trait effects

and differences in trait dispersal patterns are archived on Figshare at https://doi.org/10.6084/m9.figshare.21897003. Further data and for preliminary and intermediate analyses will be made available by the authors upon reasonable request.

Human research participants

Policy information about studies involving human research participants and Sex and Gender in Research.

Reporting on sex and gender	N/A
Population characteristics	N/A
Recruitment	N/A
Ethics oversight	N/A

Note that full information on the approval of the study protocol must also be provided in the manuscript.

Field-specific reporting

Please select the one below that is the best fit for your research. If you are not sure, read the appropriate sections before making your selection.

☐ Life sciences ☐ Behavioural & social sciences ☒ Ecological, evolutionary & environmental sciences

For a reference copy of the document with all sections, see nature.com/documents/nr-reporting-summary-flat.pdf

Ecological, evolutionary & environmental sciences study design

All studies must disclose on these points even when the disclosure is negative.

Study description	We compiled phylogenetic, trait and species distribution data for 56 clades of tetrapods (spread across 10 amphibian clades, 15 mammal clades, 17 reptile clades, and 14 bird clades). We used trait-dependent and trait-independent biogeographic models to investigate the effect of traits in clades' biogeographic histories. We further analysed differences in the magnitude of trait effects and trait-dispersal patterns between clades.
Research sample	We compiled species-level trait data, species distribution data and phylogenetic data for 56 clades of tetrapods (7009 species spread across 10 amphibian clades, 15 mammal clades, 17 reptile clades, and 14 bird clades). These clades were chosen based on data availability. Trait data contained body size and life history traits. Species distribution data contained polygons of ranges and point data. Phylogenetic data was compiled in form of dated phylogenetic trees. Data sources are listed in the methods section of the main manuscript and in the extended data files.
Sampling strategy	N/A
Data collection	Data were collected from publicly available sources by the first author with help from the third author.
Timing and spatial scale	Data were compiled from publicly available sources between 04/2020 and 04/2021.
Data exclusions	No data were excluded.
Reproducibility	We have provided data and code to repeat our analyses.
Randomization	Randomization was not applicable since our study is not experimental.
Blinding	Blinding was not applicable since our study is not experimental.

Did the study involve field work? ☐ Yes ☒ No

Reporting for specific materials, systems and methods

We require information from authors about some types of materials, experimental systems and methods used in many studies. Here, indicate whether each material, system or method listed is relevant to your study. If you are not sure if a list item applies to your research, read the appropriate section before selecting a response.

Materials & experimental systems

n/a	Involved in the study
☒	☐ Antibodies
☒	☐ Eukaryotic cell lines
☒	☐ Palaeontology and archaeology
☒	☐ Animals and other organisms
☒	☐ Clinical data
☒	☐ Dual use research of concern

Methods

n/a	Involved in the study
☒	☐ ChIP-seq
☒	☐ Flow cytometry
☒	☐ MRI-based neuroimaging
